# Knowledge, Attitude, and Practice towards Malaria among People Attending Mekaneeyesus Primary Hospital, South Gondar, Northwestern Ethiopia: A Cross-Sectional Study

**DOI:** 10.1155/2021/5580715

**Published:** 2021-12-23

**Authors:** Belaynesh Tazebew Flatie, Abaineh Munshea

**Affiliations:** Biology Department, Science College, Bahir Dar University, Ethiopia

## Abstract

**Background:**

Malaria is one of the most severe public health problems worldwide. It is a leading cause of suffering, death, and socioeconomic problem, especially in many developing countries like Ethiopia. To introduce appropriate preventive and control measures, assessment of community's levels of knowledge, attitude, and preventative practices regarding malaria is crucial. This study was aimed at assessing the knowledge, attitude, and practice (KAP) towards malaria and its preventive and control methods among people attending Mekaneeyesus primary hospital, South Gondar, northwestern Ethiopia.

**Methods:**

A hospital-based cross-sectional study was conducted from September 2017 to April 2018. A structured questionnaire was administered to collect data on sociodemographic characteristics and KAP of 390 randomly selected individuals. The data collecting tool was pretested before commencing the actual data collection. The data were analyzed using the SPSS version 21 software. *P* values less than 0.05 were considered statistically significant.

**Results:**

The overall prevalence rate of malaria in the study area was 8.5%. Nearly two-third of the participants had good knowledge (63.1%) and positive attitude (62.6%) scores towards malaria while only half of the participants had (50.8%) good practice score towards malaria prevention and control measures. Sex, age category, family monthly income, residence, and occupational and educational status of the participants were significantly associated with knowledge and practice scores (*P* < 0.05). The odds of malaria were 26.93 (CI = 3.67‐197.47, *P* = 0.001) and 13.09 (CI = 0.93‐183.47, *P* = 0.036) times higher among individuals who had poor knowledge and poor practice towards malaria, respectively, as compared to individuals who were knowledgeable and had good practice score towards malaria.

**Conclusion:**

The overall knowledge score, attitude, and practice level of respondents towards malaria was relatively good. However, significant proportion of the participants still have misconception about the cause, sign and symptoms, modes of transmission, and practices towards prevention methods of malaria. Thus, health education which is aimed at raising community's awareness about the disease is necessary to address the gaps identified by this study.

## 1. Introduction

Malaria is a serious mosquito borne infectious disease caused by an obligate intracellular protozoan parasite of the genus *Plasmodium*. There are five *Plasmodium* species causing malaria in human, *P. falciparum*, *P. vivax*, *P. malaria*, *P. ovale*, and *P. knowlesi. P. falciparum* and *P. vivax* account for more than 95% of the cases of malaria worldwide. *P. falciparum* is fatal in its characteristics and responsible for most of the malaria overall deaths [1].

Despite being preventable and curable, malaria continues to have a devastating impact on people's health and livelihoods around the world. According to the latest world malaria report, there were an estimated 229 million malaria cases and 409,000 deaths globally in 2019 [2]. Above and beyond such a huge health consequence, malaria imposes a heavy economic burden on individuals, households, and the entire economy [3]. Sub-Saharan African countries carried a disproportionately high share of the global malaria burden and accounted for 94% of all malaria cases and deaths [2].

In Ethiopia, about 75% of the land mass is malarious and 68% of the country's population is at risk of malaria due to climatic and ecological conditions favorable for its transmission. Around 4 up to 5 million cases of malaria and 70,000 related deaths are reported annually in Ethiopia [4]. Malaria still remains to be a leading public health problem and has been one of the main causes of hospitalization and deaths in the country [5, 6].

Malaria can easily be prevented through individual and societal combined efforts by keeping the environment safe, effective utilization of long lasting insecticide nets (ITNs), and early diagnosis and prompt treatment [7, 8]. Widespread control and elimination measures were implemented through international and national malaria control programs; however, malaria continues to be the most important parasitic disease worldwide. In Ethiopia, the key malaria control strategies are prompt diagnosis and immediate treatment of cases. Besides these, there are other strategies like outbreak investigation and arrest, mosquito vector control, and environmental management. Indoor residual sprays and insecticide-treated nets are also used at a large [9].

The scope of malaria control is changing worldwide with more emphasis is being placed on community and individual participation in malaria control and prevention measures than on exclusive use of insecticides. In this regard, understanding the level of knowledge, attitude, and practices of individuals and communities towards the disease and its risk factors is crucial to ensure appropriate intervention measures [10]. Health education can improve participation in malaria control, when such education is designed to address gaps in the knowledge, attitudes, and practice of individuals in the communities [11, 12].

Knowledge, attitude, and practice (KAP) studies are a useful method to design and execute malaria prevention and control programs. Several studies have shown that improving community's knowledge, attitudes, and practices can play an effective role in controlling the spread and reducing the burden of malaria [13, 14].

Varying reports regarding the knowledge about malaria in different parts of Africa and around the world reveal that gap in the knowledge about the malaria disease condition may lead individuals not to actively participate in the control programs [15]. The knowledge of the community is far from perfect, and misconceptions are rampant. Despite reasonable knowledge on malaria and its preventive measures, there is a need to improve availability of information through proper community channels. Special attention should be given to illiterate community members. High acceptance of indoor residual spraying and high level of bed net ownership should be taken as an advantage to improve malaria control [16].

Many of the human behaviors favor malaria transmission stem from broad social, cultural, and economic forces. In addition to these broad social forces, malaria transmission and control are invariably affected by local beliefs, attitudes, and practices [17]. It is clear that a change in behavior is an important component in malaria prevention and control, but the basis of the behavior elucidated by determining the levels of malaria knowledge, attitude, and practices of the community is even more crucial [15]. Knowledge in malaria reinforces the capacity of the host to affect transmission intensity through informing attitudes and behavior [18].

There have been a considerable number of reports about knowledge, attitudes, and practices relating to malaria and its control from different parts of Ethiopia. These reports concluded that misconceptions about the cause and transmission of malaria still exist, and that practices for the control of malaria have been inadequate [19–25]. In this direction, in order to create awareness in particular community, insight into the gaps of knowledge, attitudes, and practices regarding malaria is important. As well, to the best of our knowledge, no study has been conducted about the malaria knowledge, attitudes, and practices (KAP) in the population of Mekaneeyesus, Estie District, South Gondar, northwestern Ethiopia. Thus, this study was aimed at investigating knowledge, attitude, and practice towards malaria among people attending Mekaneeyesus primary hospital, South Gondar, northwestern Ethiopia (a cross-sectional study).

## 2. Materials and Methods

### 2.1. Study Area and Design

A cross-sectional study was conducted from September 2017 to April 2018 to evaluate the levels of knowledge, attitudes, and practices towards malaria among people attending Mekaneeyesus primary hospital, South Gondar, northwestern Ethiopia. Mekaneeyesus is the capital of Estie District. Estie is one of the 105 districts in Amhara Regional state of Ethiopia. Geographically, the study area lies on the coordinates of 11°34′N, latitude and 36°41′E, and longitude and at an altitude range of 1500-4000 meters above sea level (m.a.s.l). The minimum and maximum mean annual rainfall of the area is 1307-1500 mm, and the mean annual minimum and maximum temperature is 8.3°C-25°C. The district exhibits four climate zones: Wurch (upper highlands above 3,200 m a.s.l), Dega (highlands 2,300–3,200 m a.s.l), Woinadega (midlands 1500–2,300 m a.s.l), and Kola (lowlands 500–1500 m a.s.l). The peak times of malaria transmission occur between September and December following the main rainy season from June to August and from April to June.

Estie is about 676 km northwest of Addis Ababa, capital city of the country, and about 110 km north of Bahr Dar, the regional capital. The total area of this district is 132,373.9 km^2^. It has 42 rural and 3 urban kebeles (small administrative unit). Based on figures published by the Central Statistical Agency (CSA) in 2005, Estie has an estimated 403,956 population, of whom 199,325 are men and 204,631 are women; 16,014 (3.96%) of its population are urban dwellers.

### 2.2. Sample Size Determination and Sampling Techniques

The sample size of the study was determined using single population proportion formula by taking the proportion with confidence interval at 95% and alpha at 5% [26].

(1) $$\begin{array}{c} n=\frac{z^{2}p\left(1-p\right)\;}{d^{2}}, \end{array}$$

where *n* is the sample size, *z* is the *z* statistic for a level of confidence (*z* = 1.96 at 95% CI), *d* is the precision (if 5%, *d* = 0.05), and *p* is the proportion of malaria prevalence (*p* = 0.5) which was considered, since there were no similar previous studies in the study areas. Accordingly, the sample size of the study was

(2) $$\begin{array}{c} =\frac{3.8416\times 0.5\times 0.5\;}{0.0025}=384.16\approx 384. \end{array}$$

In addition, 5% nonresponse rate was added for individuals who fail to participate in the study. Then, the final sample size of the study was 403. Based on these assumptions, the study participants were recruited using simple random sampling technique.

### 2.3. Inclusion and Exclusion Criteria

Individuals who consented to participate were included in this study, while individuals who cannot communicate due to impairment or severely sick and mentally sick people and those who did not provide consent and assent were excluded from the study.

### 2.4. Data Collection

#### 2.4.1. Questionnaire Survey

A standard KAP questionnaire was compiled and adapted from similar previous studies to collect data on sociodemographic characteristics, knowledge and attitude of the study participants about malaria, its transmission, symptoms, preventive measures, and practices towards ITN ownership and use. The questionnaire was first developed in English and translated into the local language, Amharic, and then translated back to English to check consistency and phrasing of difficult concepts.

The knowledge scores of the participants towards malaria were determined as follows. The response to each correct knowledge question was given a score of 1 while a wrong or unsure response was scored as 0. The original Bloom's cut-off points where a score of 80.0%–100.0% of correct responses meant a good knowledge, a score of 60.0%–79.0% put a scorer in a level of satisfactory knowledge, and a poor knowledge was for the respondents with a score ≤ 59.0% of the correct responses were adapted and modified. Attitude was assessed by Likert's scaling technique. The questions on Likert's scaling had positive and negative responses that ranged from strongly agree (score 5), agree (score 4), undecided (score 3), disagree (score 2), to strongly disagree (score 1). The responses were summed up, and a total score was obtained for each respondent. The mean score was calculated, and respondents with score of greater than or equal to the mean score (4.14) were considered as having positive attitude while those with score of less than the mean score (4.14) were taken as having negative attitude towards malaria.

Practices of the participants were also determined using Likert's scaling method. The scoring system of Likert's type scales with respect to respondents response ranging from never (score 0), sometimes (score 1), to always (score 2) were used. The responses were summed up, and total score was obtained for each respondent, and mean practice score was computed across all the study participants. An individual was claimed as having good practice when his/her overall practice score (1.03) was equal to or more than the mean practice score. However, an individual was considered as having poor practice when his/her overall practice score is less than the mean practice score (1.03).

#### 2.4.2. Laboratory Examination and Parasite Detection

Capillary blood samples were collected by finger pricking using 70% isopropanol and sterile disposable lancet. Immediately, thin film was spread on grease free, frosted end of labeled slide using a smooth edged slide spreader. The thick smear was also prepared on the same slide by spreading larger drop of blood. The thin blood smear was allowed to air dry for 10 minutes and then fixed with absolute methanol for 5 seconds and then air-dried. The thick smears were air-dried for about 30 minutes, not fixed in methanol but dipped in water to dehaemoglobinize. The blood films were stained with 10% Giemsa for 10 minutes [27]. Finally, the films were examined under the microscope using an oil immersion microscope objective (100x) for detection of *Plasmodium* spp.

### 2.5. Data Analysis

The data gathered were double entered in to Microsoft Excel data sheets and were crosschecked and imported into SPSS version 21 for analysis. Descriptive statistics was carried out to measure relative frequencies and percentages of the variables. Frequency distribution tables were used to present sociodemographic variables, knowledge and attitude of respondents related to symptoms, causes, transmission, prevention and control measures of malaria, and practices towards malaria prevention and control methods.

Logistic regression analysis was performed to examine associations between variables by using odds ratio. Variable having significance at *P* values 0.25 in univariate test was selected and entered for multivariate logistic regression analysis to identify the most important predictors of malaria risk [28]. Odds ratios (ORs) were calculated with 95% confidence interval (CI). *P* values less than 0.05 were considered statistically significant.

### 2.6. Data Quality Control

The questionnaire was compiled and adapted from similar previous malaria indicator survey questionnaires. Before going to the actual data collection, the questionnaire was pretested to ensure the validity of the data collection tool. A pretest was carried out on 19 individuals (5% of the calculated sample size) that were not part of the sample population in the actual study at Mekaneeyesus hospital. The data gathered were double entered in to Microsoft Excel data sheets and were crosschecked and imported into SPSS version 21 for analysis.

### 2.7. Ethical Consideration

The study protocol of the research was reviewed and approved by the Ethical Review Committee under the research and community service coordinating office of College of Science, Bahir Dar University. Mekaneeyesus primary hospital granted permission for the study to be conducted at the target outpatient department after explaining the objective of the study. Informed written and oral consent was also obtained from the study participants before interview. For those who did not read and understand the consent form, the objectives of the study were explained to them and verbal consent was obtained. For children less than 18 years old, consent was obtained from their parents or guardians.

## 3. Result

### 3.1. Sociodemographic Characteristics

Of the total 403 individuals invited, 390 (97.0%) participated in the study and the remaining 13 (3.0%) individuals who refused to participate were excluded from the study. About 211 (54.1%) of the participants were males, and the remaining 179 (45.9%) were females. The mean age of the sampled population was 31.48 ± 15.62 years, and the highest number of participants (108 (27.7%)) was within the age range of 25-34 years. More than half (242 (62.1%)) of the respondents were urban residents, and majority (186 (47.7%)) were married. The educational background of the study participants varied from those who were illiterate to those who attained the levels of college and above (Table 1).

### 3.2. Knowledge about Malaria

Amharic version of malaria is known as “webba,” which is the most commonly used term in the study areas. Most of the participants (336 (86.2%)) attributed a mosquito bite as the cause of malaria. Misconceptions regarding the causes of malaria were also reflected in 10 (2.6%) of the subjected who related it with flies, and surprisingly, 44 (11.3%) of the participants did not know the cause of malaria. The majority (320 (82.1%)) of respondents had knowledge how malaria is transmitted, and more than three quarter (294 (75.4%)) of the study subjects implicated anopheles mosquitoes in the transmission of malaria. However, few participants had misconceptions about the mode of malaria transmission, and the remaining 66 (16.9%) did not know how malaria is transmitted (Table 2).

Two hundred sixty-five (67.9%), 80 (20.5%), 41 (10.5%), and 4(1.0%) of the study participants mentioned that stagnant water, tall grass, bushes, and running water can serve as breeding sites of mosquitoes, respectively. Most (354 (90.8%)) of the respondents identified that mosquitoes bite during night time. Almost all respondents identified the major sign and symptoms of malaria correctly; 386 (99.0%), 382 (97.9%), 383 (98.2%), and 377 (96.7%) mentioned fever, headache, chill and shivering, and loss of appetite as symptoms of malaria, respectively. In the present study, 256 (65.6%) and 251 (64.4%) of study participants identified underfive children and pregnant women as the most susceptible segments of the population to malaria, respectively (Table 2).

In response to knowledge about indoor preventing methods of malaria, most of the respondents (218 (55.9%)) considered using insecticide-treated bed net (ITN) as the best indoor intervention method for preventing and controlling the disease, while only 21.8% of respondents believed that malaria can be prevented using indoor residual spray (IRS), and few of them mentioned fumigation (3(0.8%)) as one of the preventive measures. Regarding the knowledge of the study participants towards the outdoor preventive methods of malaria, majority, 85.6 and 93.1%, of them mentioned that malaria can be prevented by avoiding weeds and stagnant water, respectively, whereas about one in ten (9.5%) mentioned the use of insecticide spray (Table 2).

**Figure 1:**
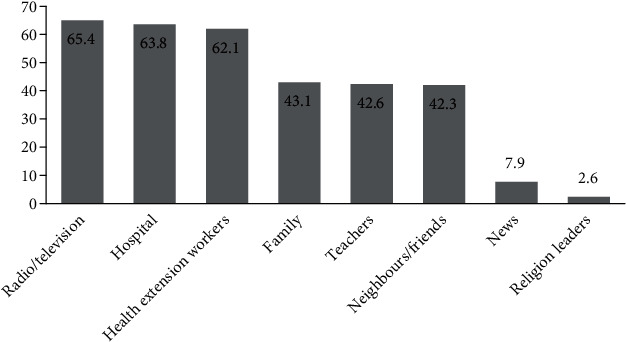
Sources of information about malaria as reported by respondents in Mekaneeyesus hospital, 2018.

The respondents of this study had information about malaria from varied sources, of which 255 (65.4%) received information through radio/television, 249 (63.8%) from hospital, 242 (62.1%) from health extension workers, 168 (43.1%) from family, 166 (42.6%) from teachers, and 165 (42.3%) from neighbors/friends (Figure 1).

### 3.3. Attitudes towards Malaria

From the total respondents, 361 (92.5%) agreed the seriousness and threat posed by malaria. The larger proportion (320 (82.2%)) of the subjects agreed that malaria is a preventable disease. The vast majority of participants (351 (90%)) felt worried when mosquitoes are nearby. Most (382 (97.9%)) of the study participants mentioned that they sleep under a mosquito net during the night to prevent themselves from mosquito bite. On the other hand, 360 (92.3%) of respondents agreed on risk incurred when malaria medicine is not taken properly and completely; however, about 4 (1%) and 26 (6.4%) of the study participants disagreed and remain neutral to this statement, respectively (Table 3).

### 3.4. Practices towards Malaria

Interestingly, majority of the study participants (320 (82.1%)) responded that they always visit health center/clinic when they or their family members get sick. More than half (216 (55.4%)) of the study participants reported that they always sleep under insecticide-treated mosquito nets, and 170 (43.6%) had the habit of checking the presence of holes/repair in the mosquito nets to avoid the mosquito bite. Nobody (0%) had the practice of constantly draining standing water where anopheles mosquito may breed. Of the total respondents, 268 (68.7%) reported that they sometimes drain stagnant water or moist areas around their residence, while 248 (63.6%) of the respondents preferred trimming bushes where mosquitoes rest and hide during the daytimes (Table 4).

**Figure 2:**
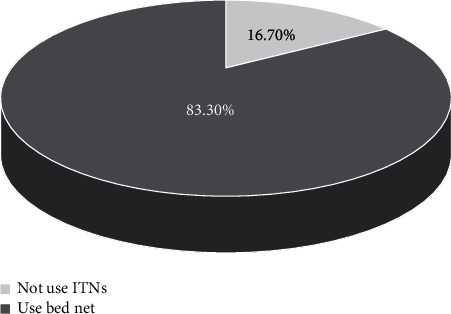
Practice of using ITN among the respondents in Mekaneeyesus hospital, 2018.

This study revealed that the majority (325 (83.3%)) of respondents had the practice of utilizing bed net while the remaining 65 (16.7%) had no the practice of using ITN. Of total number of the study participants who did not use ITN, about1 0.3% reported that they lack awareness about the use of ITN, while majority of them (64 (16.7%)) reported unavailability of ITN in local markets (Figure 2).

### 3.5. Overall Knowledge, Attitude, and Practice (KAP) Scores of the Study Participants and Its Association with Prevalence of Malaria

Assessment of knowledge score revealed that 246 (63.1%), 92 (23.6%), and 52 (13.3%) of the study participants had good (knowledgeable), satisfactory, and poor knowledge about malaria, respectively. With regard to attitude, nearly two-third (62.6%) of the study participants had positive attitude while the remaining146 (37.4%) had negative attitude towards malaria in terms of its seriousness or threat, prevention, and control. The study participants' overall practice score towards malaria prevention and control was calculated, and practice level was determined by comparing one's practice score against the mean practice score. Accordingly, nearly equal proportion of study participants had good (50.8%) and poor (49.2%) practice towards malaria prevention and control measures (Table 5).

Out of the 390 microscopically examined blood samples, 33 samples were found positive for malaria infection with the overall prevalence rate of 8.5%. In Chi-square analyses, the prevalence of malaria was significantly associated with the overall KAP scores of the study participants (*P* < 0.05) (Table 5).

### 3.6. Associations of Knowledge and Practice Scores of the Study Participants with Sociodemographic Status

In Chi-square analysis, marital status was not significantly associated with knowledge score of the respondents (*P* = 0.129). However, the other sociodemographic and environmental risk factors were significantly associated with knowledge score of the respondents towards malaria (Table 6).

Females were more knowledgeable (69.8%) about malaria than their male counterparts (57.3%). Similarly, significantly higher proportion of females had good practices (57.0%) towards malaria than males (45.5%). Conversely, significantly higher proportion of males (54.5%) had poor practices towards malaria than females (43.0%) (Table 6).

There was a statistically significant association between residence and knowledge score of the participants about the cause, sign and symptoms, modes of transmission, and prevention of malaria (*P* = 0.001). In this study, most (75.2%) of urban dwellers had good knowledge score about malaria than their rural counterparts (43.2%), while poor knowledge score about malaria was more evident among rural (24.3%) than urban residents (6.6%). Similarly, there was a statistically significant association between residence and practice score of respondents towards malaria treatment, prevention, and control measures (*P* = 0.001). Majority (61.2%) of urban residents had good practices towards malaria than rural (33.8%) residents; on the contrary, larger proportion (66.2%) of participants from rural setting had poor practices towards malaria than urban (38.8%) residents (Table 6).

Educational status of the study participants was significantly associated with the knowledge and practice scores towards malaria (*P* = 0.05). There was an increasing tendency in good knowledge and practice scores as educational statuses of the study participants go from uneducated to college and above. The highest good knowledge scores were recorded among those who attained high school (82.9%) and college and above (84.3%) education, who also had the lowest poor knowledge scores, 4.9% and 2.5%, respectively. With regard to practices of respondents towards malaria, the highest good practice score was observed among participants who attained college and above (74.2%) education followed by those who attained secondary (41.5%) and primary school (28.6%) education, while the lowest good practice score towards malaria was recorded among uneducated (20.2%) subjects (Table 6).

This study found a statistically significant association between family monthly income and knowledge and practice scores of the participants towards malaria (*P* < 0.05). Individuals with the highest monthly income, 2000 and above Ethiopian birr per month, had the highest of good knowledge (84.3%) and good practice score (70.4%), whereas participants with lowest monthly income had the lowest good knowledge (37.8%) and good practice scores (23.0%) towards malaria (Table 6).

### 3.7. Multivariate Logistic Regression Analysis of Malaria Prevalence with KAP Scores

In multiple regression analysis, the odds of malaria was significantly twenty seven times higher in individuals who had poor knowledge (AOR = 26.93, 95% CI 3.67-197.47, and *P* = 0.001) than those who had good knowledge, while statistically nonsignificant three times (AOR = 2.97, 95% CI 0.51-17.46, and *P* = 0.228) increased risk of malaria infection was detected among participants with satisfactory knowledge score as compared to those with good knowledge score. Prevalence of malaria did not show any significant association with regard to attitude levels of the present participants. On the other hand, the odds of positive malaria diagnosis was thirteen times higher in those who had poor practice than those who had good practice, and it was statistically significant (AOR = 13.09, 95% CI 0.93-183.47, and *P* = 0.036) (Table 7).

## 4. Discussion

Of the total 390 individuals participated in this study, majority (82.1%) mentioned that they know how malaria is transmitted, and 86.2% and 75.4% of them associated mosquito bite as a cause of malaria and means of disease transmission, respectively. This observation supports finding of another study conducted in Tanzania, which reported that more than 80% of participants had knowledge about malaria transmission [29]. This finding is also comparable with the reports of the studies in Swaziland [30], Northwest Tanzania [16], India [31], and Mexico [32]. However, finding of this study is higher than the one reported in India [10] and Nigeria [33]. Contrary to this finding, study conducted in Shashogo District of Ethiopia reported a very low knowledge level of respondents about the mode of malaria transmission where only 15.6% of the participants associated mosquitoes with malaria [34]. Besides, studies conducted in Ethiopia such as in Assosa Zone, Western Ethiopia, found that less than half (47.5%) of the study participants mentioned mosquito bites as a mode of malaria transmission, and thirty percent (30%) of them were aware that mosquitoes are the carriers of disease causing microorganism [10], and a report in Amhara region, Ethiopia, revealed that 32.3% of the study participants implicated mosquito bite in transmission of malaria [17].

In present study, almost all respondents identified the major sign and symptoms of malaria correctly; 386 (99.0%), 382 (97.9%), 383 (98.2%), and 377 (96.7%) mentioned fever, headache, chill and shivering, and loss of appetite as symptoms of malaria, respectively. This finding is comparable with a finding of study conducted in Karachi [35] and two other studies conducted in Ethiopia [10, 36].

Knowledge about mosquito behaviors, resting and breeding places and feeding time, is important to take appropriate malaria preventive actions and proper use of ITNs. Observations regarding breeding sites of mosquitoes showed that 265 (67.9%) of the study participants associated it with stagnant water. The result is consistent with some other study done in India [37] and in Shashogo District, Southern Ethiopia [34]. In our study, the proportion of subjects who knew stagnant water as mosquito breed site is lower as compared with a report of a study conducted in Tepi Town, Sheka zone, Southwestern Ethiopia, in which most (96.4%) of the community members were aware that the mosquito breeds in stagnant water [38]. However, the present result is higher than a finding reported from India, where less than half (32.7%) of the respondents knew that mosquitoes most commonly breed in stagnant water [31].

It was also observed that most (354 (90.8%)) of participants in our study identified that mosquitoes bite during night time. This is similar with what was reported in Assosa Zone, Western Ethiopia, where most (95%) of respondents replied that mosquitoes bite in the night [10]. Participants' knowledge about mosquitoes feeding time in this study is encouraging when compared to 56.5% report from India [31].

In the present study, 256 (65.6%) and 251 (64.4%) of study participants identified correctly underfive children and pregnant women as the most susceptible group of the population to malaria, respectively. This result concurs with the findings of similar studies conducted in Kenya [8] and Southwestern Ethiopia [30]; in both cases, significant proportion of the participants identified underfive children and pregnant women as the most vulnerable segment of the population to malaria. This is mainly due to the fact that children under five years of age have less developed and weak immunity that makes them more vulnerable to diseases compared to adults and pregnant women have semicompromised immunity.

In this study, 361 (92.5%) of the respondents agreed the seriousness and threat posed by malaria and 320 (82.2%) of the subjects agreed with the statement that malaria is preventable disease, which is comparable with the study conducted in Shewa Robit, Ethiopia, in which about 90.58% respondents believed that malaria is preventable disease [36].

It was also revealed that the majority (325 (83.3%)) of respondents of the current study had the practice of utilizing bed net while the remaining 65 (16.7%) had no the practice of using ITN. Of total number of the study participants who did not use ITN, about 0.3% reported that they lack awareness about the use of ITN, while majority of them (64 (16.7%)) reported unavailability of ITN in local markets. However, nobody reported the expensiveness of ITNs. ITN utilization practice coverage observed in our study is in agreement with 85 and 78% bed net utilization results reported in Karachi [35] and in Swaziland [30], respectively. The rate of ITN utilization practice in our study is slightly lower compared to a finding of a study in Colombia, where most of the study population (>90%) had a practice of using ITNs [39]. The bed net utilization practice observed in the current study is also relatively similar with the one reported from Southern Mexico, in which most (76%) used them bed net all year round [32]. However, these respondents did not associate bed net utilization with malaria prevention rather with protection against mosquito bite. Another study conducted in Southwestern Ethiopia reported 65.0% bed net utilization rate, which is far less than our finding. Of the remaining 35.0% of the participants who did not use bed net, most (77.0%) replied that they were not lucky to use bed nets due to lack of access, 8.0% associated their failure to use bed net with lack of awareness, and the remaining 15.0% suggested other reasons [38]. The difference in bed net utilization rates across studies and communities might be due to variations in monthly income, availability of bed net in markets, and unevenness in distribution of bed net, and in some cases, it may due to differences in awareness among the studied communities [40].

Nearly two-third (63.1%) of the study participants had good knowledge score about malaria. This is lower when compared with a study from Southern Ethiopia, where 74.3% of respondents had good knowledge [36]. However, it is encouraging when compared to finding of a study conducted in Champasack Province, Lao PDR, where 59.1% of respondents had good knowledge [41]. Likewise, a report of a study from Mumbai, India, revealed that 53.7% respondents had an average level and very few have high level of knowledge [42]. Differences in demographic, socioeconomic, educational, and cultural factors among communities and the absence, inaccessibility, or inaccuracy of information about the disease could affect knowledge scores.

This study revealed that 244 (62.6%) of the study participants had positive attitude while remaining 146 (37.4%) had negative attitude towards malaria in terms of its seriousness or threat, prevention, and control. This is lower when compared with the reports of 97.0% and 78.1% positive attitudes of the participants towards malaria prevention in Karachi [35] and in Amhara National Regional State of Ethiopia [17], respectively.

In this study, half (50.8%) of the study participants had good practice score towards malaria prevention and control measures. Similar studies done in Sri Lanka [43] and Southern Ethiopia [34] reported fairly good practice towards malaria prevention and control measures. Our result is lower when compared to the finding of another study conducted in Southern Ethiopia, where 67.7% of the study participants had good practice in terms of malaria treatment, prevention, and control [10] and a study from Karachi that reported 59% good practice [35]. Conversely, a study conducted among population in Paksong District, Champasack Province, LAO PDR, found only 5.7% good practice regarding malaria prevention [41]. These discrepancies in implementation of good practices towards malaria prevention and control might be due to differences in sociodemographic characteristics (gender, age, educational, and income levels), the community's awareness about malaria, and their attitudes towards malaria prevention and control.

In this study, female participants were found to be more knowledgeable (69.8%) about malaria than their male counterparts (57.3%), suggesting a need for awareness creation towards malaria for the males. This is supported by study conducted in Ha-Lambani, Limpopo Province, South Africa [44], which also reported that more female participants had knowledge on malaria transmission and symptoms than males. Similarly, significantly higher proportion of females (57.0%) had good practices towards malaria than males (45.5%) (*P* = 0.05). Conversely, considerably higher proportion of males (54.5%) had poor practices towards malaria than females (43.0%). This is in conformity with the finding that reported statistically significant association between practice level and gender of participants, in which greater proportion (45.0%) of female participants had good practice towards malaria than males (28.1%); contrarily, substantially higher percentage (71.9%) of male participants had poor practice level towards malaria as compared with female participants (55.0%) [45]. This could be due to the fact that women in developing countries mainly take the role of looking after their family members. Conversely, our finding does not agree with the findings of studies conducted in Ethiopia [46] and Cabo Verde [47].

Participants in age categories 15-24 and 25-34 years old had the highest good knowledge. This is in line with study done by [46]. The finding of this study contradicts with the reports of studies conducted in Cabo Verde [47] and Myanmar [48], which showed an increase in the scores of good knowledge and good practice towards malaria as the ages of the study participants' increase.

In this study, there was a significant increasing tendency in good knowledge and practice scores as educational level of the study participants increases from uneducated to college and above levels (*P* = 0.05). The highest good knowledge scores were recorded among those who attained college and above (84.3%) and high school (82.9%) education, who also had the lowest poor knowledge scores, 2.5% and 4.9%, respectively. Likewise, the highest good practice score was observed among participants who attained college and above (74.2%) education followed by those who attained secondary (41.5%) and primary school (28.6%) education, while the lowest good practice score towards malaria was recorded among uneducated (20.2%) subjects. This could be explained by the fact that illiterate people and those with low levels of education might not be able to understand and access health education information conveyed through various mass media appropriately. This could lead to poor knowledge score about malaria, which consecutively affects their action towards malaria prevention and control measures. Our findings are consistent with the studies conducted in Indonesia [49] and Cameroon [50].

This study found statistically significant association between family monthly income and knowledge and practice scores of the participants towards malaria (*P* < 0.05). Individuals with the highest monthly income, 2000 and above Ethiopian birr per month, had the highest of good knowledge (84.3%) and good practice score (70.4%), whereas participants with lowest monthly income had the lowest good knowledge (37.8%) and good practice scores (23.0%) towards malaria. This can be attributed to an increase in the family monthly income which may lead to increase in the opportunity of accomplishing supplies for protecting. The result of the present study is coincident with the reports of a study in Myanmar [48].

In this study, there was a statistically significant association between residence and knowledge score of the participants about the cause, sign and symptoms, and methods of transmission and prevention of malaria (*P* = 0.001). The finding of this study showed that living in the urban area increased the level of knowledge on malaria. Most (75.2%) of urban dwellers had good knowledge score towards malaria than their rural counterparts (43.2%), while poor knowledge score about malaria was more evident among rural (24.3%) than urban residents (6.6%). This may be due to the fact that women from urban residence may have more exposure and access for education and health-related information via mass media than those from rural areas. This finding is supported by studies done in Ethiopia and Malawi, which state that participants from urban residence were more knowledgeable than rural areas, as rural residence may hinder people to access health information and health literacy [51, 52]. However, contrary to our finding, Kimbi and his coworkers in Cameroon found no statistically significant difference in malaria knowledge level between participants from rural (98.04%) and urban (98.97%) areas [53].

In this study, the odds of malaria infection in individuals who had poor knowledge and poor practice were 26.93 and 13.09 times higher, respectively, as compared to individuals who were knowledgeable and had good practice towards malaria. Similar findings were found in the study done in south-central Ethiopia [54], but respondent's knowledge about malaria was not significantly associated with malaria risk. It is likely to argue that increased level of knowledge on malaria is associated with reduced risk of malaria. People who have a high level of knowledge are in a better position to protect them against malaria.

## 5. Conclusion and Recommendation

In this study, the overall knowledge score, attitude, and practice level of the study population towards malaria was relatively good. However, substantial proportion of the participants still have misconception about the cause, sign and symptoms, modes of transmission, and practices towards prevention methods of malaria. Thus, health education which is aimed at raising community's awareness about the disease is necessary to address the gaps identified by this study.

## Figures and Tables

**Table 1:** Sociodemographic characteristics of respondents in Mekaneeyesus hospital, South Gondar, northwestern Ethiopia, 2018.

Variable	Category	Frequency (*n*)	Percentage (%)
Sex	Male	211	54.1
	Female	179	45.9

Age category	Under 5	12	3.1
	5-14	26	6.7
	15-24	94	24.1
	25-34	108	27.7
	35-44	72	18.5
	45-54	40	10.3
	≥55	38	9.7

Marital status	Unmarried	176	45.1
	Married	186	47.7
	Widow/widower	15	3.8
	Divorced	13	3.3

Educational status	Uneducated	109	27.9
	1-8	42	10.8
	9-12	41	10.5
	College and above	198	50.8

Religion	Orthodox	273	70.0
	Muslims	95	24.4
	Protestant	20	5.1
	Catholic	2	0.5

Occupation status	Unemployed	45	11.5
	Daily laborer	30	7.7
	Student	73	18.7
	House wife	20	5.1
	Farmer	71	18.2
	Merchant	74	19.0
	Government employee	77	19.7

Residence	Rural	148	37.9
	Urban	242	62.1

Family monthly income in Ethiopian birr (ETB)	Less than 500	74	19.0
	500-1000	63	16.2
	1001-1500	43	11.0
	1501-2000	31	7.9
	Above 2000	179	45.9

**Table 2:** Knowledge of respondents related to cause, sign and symptoms, transmission of malaria, and mosquito breeding areas, Mekaneeyesus hospital, South Gondar, northwestern Ethiopia, 2018.

Variables	Categories		Frequency *n* (%)
Cause of malaria	Mosquito bite		336 (86.2)
	Flies		10 (2.6)
	Do not know		44 (11.3)

Do you know how malaria is transmitted?	Yes		320 (82.1)
	No		70 (17.9)

Malaria transmission	Mosquito bite		294 (75.4)
	Drinking untreated water and eating dirty food		13 (3.3)
	Contacting malaria patient		17 (4.4)
	Do not know		66 (16.9)

When do mosquitoes mostly bite?	During the day time		8 (2.1)
	During night time		354 (90.8)
	Any time		24 (6.2)
	I do not know		4 (1.0)

Mosquitoes breeding site	Stagnant water		265 (67.9)
	Tall grass		80 (20.5)
	Bushes		41 (10.5)
	Running water		4 (1.0)

Indoor preventing methods of malaria	Insecticide-treated bed net		218 (55.9)
	Insecticide residual spray		85 (21.8)
	Fumigation		3 (0.8)
	Keep windows and doors closed in the evening		84 (21.5)

Outdoor preventing methods of malaria	Avoid weeds	Yes	334 (85.6)
		No	56 (14.4)
	Avoid stagnant water	Yes	363 (93.1)
		No	27 (6.9)
	Insecticide spray	Yes	37 (9.5)
		No	353 (90.5)

Susceptible group for malaria	Under five	Yes	256 (65.6)
		No	134 (34.4)
	Pregnant women	Yes	251 (64.4)
		No	139 (35.6)
	Elderly	Yes	153 (39.2)
		No	237 (60.8)
	Equal for all	Yes	119 (30.5)
		No	271 (69.5)

Symptoms of malaria	Fever	Yes	386 (99.0)
		No	4 (1.0)
	Head ach	Yes	382 (97.9)
		No	8 (2.1)
	Chill and shivering	Yes	383 (98.2)
		No	7 (1.8)
	Loss of appetite	Yes	377 (96.7)
		No	13 (3.3)

**Table 3:** The study participants' response towards attitude questions, Mekaneeyesus hospital, South Gondar, northwestern Ethiopia, 2018.

Statement	Strongly disagree (1)	Disagree (2)	Undecided (3)	Agree (4)	Strongly agree (5)
	*n* (%)	*n* (%)	*n* (%)	*n* (%)	*n* (%)
Blood smear is necessary for malaria diagnosis	7 (1.8)	15 (3.8)	7 (1.8	252 (64.6)	109 (27.9)

I think the presence of mosquitoes bother you	1 (0.3)	17 (4.4)	21 (5.4)	296 (75.9)	55 (14.1)

I believe to visit health center/clinic when feel sick	0 (0.0)	5 (1.3)	3 (0.8)	296 (75.9)	86 (22.1)

Do you think malaria is preventable disease	0 (0.0)	6 (1.5)	13 (3.3)	320 (82.2)	51 (13.1)

I think malaria is a serious and life-threatening (fatal) disease	1 (0.3)	18 (4.6)	10 (2.6)	255 (65.4)	106 (27.2)

I believe sleeping under a mosquito net during the night is one way to prevent myself getting malaria	2 (0.5)	2 (0.5)	4 (1.0)	196 (50.3)	186 (47.7)

I think it is risky when malaria medicine is not taken properly and completely	0 (0.0)	4 (1.0)	26 (6.4)	293 (75.1)	67 (17.2)

**Table 4:** Participants response to good practice questions related to treatment, prevention, and control of malaria, Mekaneeyesus hospital, 2018.

Practice questions	Frequency %		
	Never (0)	Sometimes (1)	Always (2)
How do you describe your habit of visiting health center/clinic when you and any of your family members fall sick?	13 (3.3)	57 (14.6)	320 (82.1)
How often do you sleep in an insecticide-treated mosquito net (ITN)?	58 (14.9)	116 (29.7)	216 (55.4)
How do you describe your habit of checking for holes/repair mosquito nets?	40 (10.3)	180 (46.2)	170 (43.6)
All family member sleep under mosquito net	255 (65.4)	134 (34.4)	1 (0.3)
How do you describe your habit of trimming bushes around your home?	139 (35.6)	248 (63.6)	3 (0.8)
How often do you drain stagnant water/moist areas around your home?	122 (31.3)	268 (68.7)	0 (0.0)

**Table 5:** Cross tabulation of Chi-square analyses of association of overall knowledge, attitude, and practice scores of the study participants with the prevalence of malaria.

Variables	Number examined (%)	Malaria positive	Malaria negative	*χ* ^2^, *P*
Knowledge level				
Poor knowledge	52 (13.3)	22 (42.3)	30 (57.7)	93.571, 0.001
Satisfactory	92 (23.6)	8 (8.7)	84 (91.3)	
Good knowledge	246 (63.1)	3 (1.2)	243 (98.8)	
Attitude level				
Negative attitude	146 (37.4)	26 (17.8)	120 (82.2)	26.320, 0.001
Positive attitude	244 (62.6)	7 (2.9)	237 (97.1)	
Practice level				
Poor practice	192 (49.2)	31 (16.1)	161 (83.9)	28.831,0.001
Good practice	198 (50.8)	2 (1.0)	196 (99.0)	

**Table 6:** Chi-square analyses of the associations of knowledge scores and practice scores of the study participants with sociodemographic status.

Variables	Category	Knowledge score			*χ* ^2^, *P*	Practice score		*χ* ^2^, *P*
		Poor *n* (%)	Satisfactory *n* (%)	Good *n* (%)		Good *n* (%)	Poor *n* (%)	
Sex	Male Female	32 (15.2) 20 (11.2)	58 (27.5) 34 (19.0)	121 (57.3) 125 (69.8)	6.513, 0.039	96 (45.5) 102 (57.0)	115 (54.5) 77 (43.0)	5.111, 0.024

Age categories	Under 5 5-14 15-24 25-34 35-44 45-54 ≥55	6 (50.0) 7 (26.9) 7 (7.4) 11 (10.2) 9 (12.5) 6 (15.0) 6 (15.8)	3 (25.0) 8 (30.8) 19 (20.2) 28 (25.9) 18 (25.0) 6 (15.0) 10 (26.3)	3 (25.0) 11 (42.3) 68 (72.3) 69 (63.9) 45 (62.5) 28 (70.0) 22 (57.9)	28.250, 0.005	1 (8.3) 5 (19.2) 44 (46.8) 62 (57.4) 46 (63.9) 22 (55.0) 18 (47.4)	11 (91.7) 21 (80.8) 50 (53.2) 46 (42.6) 26 (36.1) 18 (45.0) 20 (52.6)	26.908, 0.001

Marital status	Un married Married Divorced Widowed/widower	26 (14.8) 19 (10.2) 3 (23.1) 4 (26.7)	43 (24.4) 40 (21.5) 3 (23.1) 6 (40.0)	107 (60.8) 127 (68.3) 7 (53.8) 5 (33.3)	9.888, 0.129	72 (40.9) 116 (62.4) 7 (53.8) 3 (20.0)	104 (59.1) 70 (37.6) 6 (46.2) 12 (80.0)	22.584, 0.001

Educational status	Uneducated 1-8 9-12 College and above	31 (28.4) 14 (33.3) 2 (4.9) 5 (2.5)	51 (46.8) 10 (23.8) 5 (12.2) 26 (13.1)	27 (24.8) 18 (42.9) 34 (82.9) 167 (84.3)	131.946, 0.001	22 (20.2) 12 (28.6) 17 (41.5) 147 (74.2)	87 (79.8) 30 (71.4) 24 (58.5) 51 (25.8)	94.146, 0.001

Occupational status	Student Daily laborer Unemployed House wife Farmer Merchant Government employee	12 (16.4) 1 (3.3) 10 (22.2) 2 (10.0) 20 (28.2) 6 (8.1) 1 (1.3)	12 (16.4) 15 (50) 13 (28.9) 5 (25.0) 29 (40.8) 12 (16.2) 6 (7.8)	49 (67.1) 14 (46.7) 22 (48.9) 13 (65.0) 22 (30.0) 56 (75.7) 70 (90.9)	82.858,0.001	25 (34.2) 16 (53.3) 16 (35.6) 11 (55.0) 18 (25.4) 49 (66.2) 63 (81.8)	48 (65.8) 14 (46.7) 29 (64.4) 9 (45.0) 53 (74.6) 25 (33.8) 14 (18.2)	67.478, 0.001

Residence	Rural Urban	36 (24.3) 16 (6.6)	48 (32.4) 44 (18.2)	64 (43.2) 182 (75.2)	44.390, 0.001	50 (33.8) 148 (61.2)	98 (66.2) 94 (38.8)	27.531, 0.001

Family monthly income in Ethiopian birr (ETB)	Less than 500 500-1000 1000-1500 1500-2000 2000 and above	23 (31.1) 9 (14.3) 8 (18.6) 6 (19.4) 6 (3.4)	23 (31.1) 28 (44.4) 15 (34.9) 4 (12.9) 22 (12.3)	28 (37.8) 26 (41.3) 20 (46.5) 21 (67.7) 151 (84.3)	86.580, 0.001	17 (23.0) 18 (28.6) 17 (39.5) 20 (64.5) 126 (70.4)	57 (77.0) 45 (71.4) 26 (60.5) 11 (35.5) 53 (29.6)	67.384, 0.001

**Table 7:** Multivariate logistic regression analysis of malaria prevalence with KAP.

Variable	*N* (%)	*n* (%)	*Β*	SE	Crude OR (95% CI)	Adjusted (OR 95% CI)	*P* value
Knowledge score							
Poor	52 (13.3)	22 (42.3)	3.29	1.02	59.40 (16.77,210.35)	26.93 (3.67, 197.47)	0.001^∗^
Satisfactory	92 (23.6)	8 (8.7)	1.09	0.90	7.71 (2.00, 29.75)	2.97 (0.51, 17.46)	0.228
Good^a^	246 (63.1)	3 (1.2)			1.00	1.00	
Attitude level					
Negative	146 (37.4)	26 (17.8)	0.79	0.82	7.34 (3.09, 17.39)	2.22 (0.44, 11.12)	0.330
Positive^a^	244 (62.6)	7 (2.9)			1.00	1.00	
Practice level							
Poor	192 (49.2)	31 (16.1)	2.57	1.35	18.87 (4.45, 80.04)	13.09 (0.93, 183.47)	0.036^∗^
Good^a^	198 (50.8)	2 (1.0)			1.00	1.00	

Note: *N*: total number of study participants; *n*: positive for *Plasmodium* infection. ^a^Reference category; COR: crude odds ratio, sig. at 0.25; AOR: adjusted odds ratio, ^∗^sig. at *P* < 0.05.

## Data Availability

The datasets used and analyzed during the current study are available upon reasonable request from the corresponding author.
